# Approaches to managing medically serious suicide attempts referred to consultation-liaison psychiatry

**DOI:** 10.1192/j.eurpsy.2026.11036

**Published:** 2026-07-31

**Authors:** R. Sánchez-González, E. Monteagudo-Gimeno, E. Marí-Cardona, L. Pintor-Pérez

**Affiliations:** 1 Department of Psychiatry., Institut de Salut Mental, Centre Emili Mira. Hospital del Mar., Barcelona; 2 Benito Menni Mental Health Care Complex., Sant Boi de Llobregat; 3 Faculty of Medicine and Life Sciences. Universitat Pompeu Fabra (UPF).; 4 Consultation-Liaison Service. Department of Psychiatry., Institut de Neurociències. Hospital Clínic i Provincial de Barcelona. Institut d’Investigacions Biomèdiques August Pi i Sunyer (IDIBAPS) – Universitat de Barcelona., Barcelona, Spain

## Abstract

**Introduction:**

The term medically serious suicide attempts (MSSA) is used to group those attempts that would have been fatal if not for medical intervention. Although studying MSSA is crucial for understanding the nature of suicide and developing prevention strategies, little is known about the underlying risk factors and demographic characteristics associated with it.

**Objectives:**

To describe and evaluate the characteristics of MSSA within the context of a general hospital consultation-liaison psychiatry (CLP) service.

**Methods:**

Longitudinal observational case-control design, including adult inpatients consecutively referred to the consultation-liaison psychiatry (CLP) service at the University Clinical Hospital of Barcelona from 2007 to 2014. Cases were selected according to the criteria established by Beautrais in prior research for defining MSSA. The control group consisted of patients referred to the CLP service from medical or surgical units for reasons other than MSSA. All patients underwent assessment through a structured psychiatric interview based on the DSM-IV (SCID-CV). Data were collected prospectively via a computerized clinical database and included the following variables:

Sociodemographic factors: age, gender, and physical disability.

Psychiatric morbidity classified according to DSM-IV-TR categories, based on the SCID-CV assessment, along with history of psychiatric disorders and prior suicide attempts.

Psychosocial stressors and recent exposure to stressful life events.

Variables related to the current hospitalization and CLP intervention.

**Results:**

The study comprised 223 consultations involving patients with MSSA (4.1%) and 5,205 consultations for other reasons (95.9%). The sociodemographic and clinical characteristics of both subgroups are illustrated in the accompanying figure. Patients presenting with MSSA were significantly younger than those in the control group (47.3 vs. 55.3 years; p < 0.001). Additionally, they exhibited a higher prevalence of psychosocial stressors (54.3% vs. 24%; p < 0.001), prior suicide attempts (54.3% vs. 3.1%; p < 0.001), and psychiatric disorders (78.9% vs. 54.5%; p < 0.001), predominantly mood and personality disorders. Intensive care was required in 10% of MSSA cases, compared to only 0.9% in the control group (p < 0.001). During hospitalization, the majority of MSSA patients (77.1%) necessitated extensive follow-up within the CLP service, involving two or more visits. The mean length of hospital stay was significantly shorter for MSSA patients compared to controls (18.4 vs. 25.3 days; p < 0.003). Nevertheless, 24.3% of MSSA patients were eventually transferred to psychiatric inpatient units following discharge from medical or surgical wards.

**Image 1:**
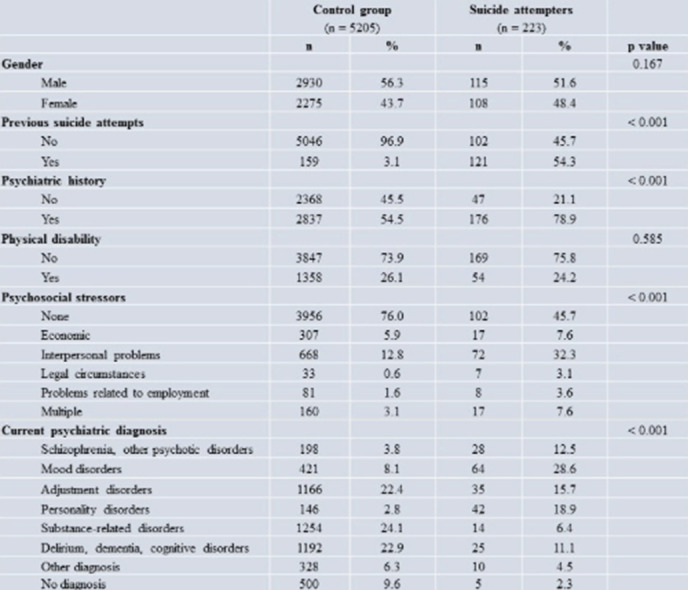
Image 1: Long description.

**Conclusions:**

Our findings corroborate that patients with MSSA referred to consultation-liaison psychiatry exhibit distinct epidemiological and clinical characteristics, enabling the identification of specific risk factors.

**Disclosure of Interest:**

None Declared

## Long descriptions

### Image 1: Long description

The table consists of seven main categorical sections with columns for Control group (n = 5205), Suicide attempters (n = 223), and p values.

* Gender: Control group is 56.3 percent male and 43.7 percent female. Suicide attempters are 51.6 percent male and 48.4 percent female. p value is 0.167.

* Previous suicide attempts: 96.9 percent of the control group had none, while 54.3 percent of suicide attempters had previous attempts. p value is less than 0.001.

* Psychiatric history: 54.5 percent of the control group and 78.9 percent of suicide attempters had a history. p value is less than 0.001.

* Physical disability: 26.1 percent of the control group and 24.2 percent of suicide attempters reported a disability. p value is 0.585.

* Psychosocial stressors: The most common stressor for suicide attempters was interpersonal problems at 32.3 percent, compared to 12.8 percent in the control group. p value is less than 0.001.

* Current psychiatric diagnosis: Suicide attempters showed higher rates of mood disorders (28.6 percent) and personality disorders (18.9 percent) compared to the control group (8.1 percent and 2.8 percent respectively). p value is less than 0.001.

Navigate back to Image 1:.

